# An enzyme free fluorescence resonance transfer strategy based on hybrid chain reaction and triplex DNA for Vibrio parahaemolyticus

**DOI:** 10.1038/s41598-020-77913-2

**Published:** 2020-11-26

**Authors:** Xiao-Hui Tan, Yu-Bin Li, Yan Liao, Hua-Zhong Liu

**Affiliations:** 1 College of Chemistry and Environmental Sciences, Guangdong Ocean University, Zhanjiang, 524088 China; 2 College of Food Science and Technology, Guangdong Ocean University, Zhanjiang, 524088 China

**Keywords:** DNA, Nucleic acids

## Abstract

In this work, an enzyme-free fluorescence resonance energy transfer (FRET) strategy was established for rapid and specific detection of the DNA sequence from *Vibrio parahaemolyticus* (VP) using hybridization chain reaction (HCR) amplification and triplex DNA. The triplex forming oligonucleotide (TFO) was labelled with carboxyfluorescein (FAM) as fluorescence donor, and hairpin sequence H1 was labelled by tetramethylrhodamine (TAMRA) as fluorescence receptor. In the present target VP DNA, the hairpin structure of molecular beacon (MB) was opened, the free end was released and hybridized with H1-TAMRA, and the HCR reaction was triggered by the alternate supplementation of H1-TAMRA and H2 to produce the notch double helix analogue. After the addition of TFO-FAM, a triplex structure was formed between HCR products (H1-TAMRA/H2) and TFO-FAM. A close contact between the donor and the receptor resulted in FRET. Under the optimal conditions, the fluorescence quenching value was inversely proportional to the concentration of target VP DNA in the range of 0.1–50 nmol L^−1^, and the detection limit was 35 pmol L^−1^.

## Introduction

Vibrio parahaemolyticus (VP) is widely distributed in seawater and seafood such as shrimp, crab, shellfish, etc., which can cause gastroenteritis, wound infection and septicemia. As a pathogenic bacteria, its clinical manifestations of infection include acute onset of abdominal pain, vomiting, diarrhea and loose stools, and even systemic spasm and acute renal failure in severe cases^1^. VP is also a common pathogen of aquatic animals. It can lead to the outbreak of red leg disease and vibrio disease in shrimp^2,3^, and infects crabs and shellfish, resulting in a large number of deaths of crabs and shellfish, seriously endangering aquaculture industry^4,5^.

In consideration of the pathogenicity of VP, a rapid and specific detection method of VP has positive implications for food safety and aquaculture. The traditional separation identification method for VP is accurate, however, it takes 5–7 days, and the operation is complex and labor-intensive, which cannot meet the needs of the rapid detection of VP. At present, many new methods for VP have been established, such as enzyme-linked immunosorbent assay (ELISA)^6^, immunofluorescence^7^, polymerase chain reaction assay^8^ and loop-mediated isothermal amplification (LAMP) detection method^9^ etc.. However, immunological detection methods are prone to false negative or false positive due to the presence of various antigens on the surface of VP. PCR detection method requires DNA polymerase. LAMP detection method has a complex system and high operational requirements. Since DNA is the basic genetic material of organisms, it is highly species-specific, which can be used as target to detect certain organisms^10^. In this work, with the purpose of rapid detection of VP, we develop an assay for the specific detection of VP DNA sequence.

As an enzyme-free amplification technique in vitro, hybridization chain reaction (HCR) has been widely used to build sensing platforms for a variety of DNA^11,12^, microRNA^13,14^, protein^15^, ion^16^, small molecule^17–19^ and cell^20–22^, and has many advantages, such as enzyme-free, mild condition and simple operation. An initiator activated HCR results in DNA self-assembly to produce double-stranded DNA (dsDNA). Moreover, HCR products can be converted into colorimetric readout^23^, fluorescence^24^, chemiluminescence^25^, electrochemical signal^26^ and other signals.

FRET is a non-radiative energy transition, transfering the energy of an excited donor state to an excited recipient state through an electric dipole interaction between molecules. The receptor absorbs the energy from the donor emission wavelength. FRET efficiency is closely correlated with the spatial distance between donor and recipient molecules^27^.

Davies and Rich discovered that when a triplex forming oligonucleotide (TFO) binds to a specific region of dsDNA, the triplex nucleic acid (triplex DNA) can be formed. Homopurine-homopyrimidine dsDNA can be identified by TFO via Hoogsteen (or reverse-hoogsteen) hydrogen bonds. As a powerful tool, triplex DNA interaction plays an important role in regulation of gene expression, construction of molecular switches for biosensors, sequence-specific recognition of the natural state of dsDNA and regulation of DNA nanostructures^28^.

Herein, an enzyme-free fluorescent sensing platform for VP DNA based on HCR amplification, FRET and triplex DNA has been developed. In the presence of VP DNA, which can hybridize with molecular beacon (MB), and the hairpin structure of MB is released. The free terminal of MB can trigger the HCR reaction, and H1-TAMRA and H2 are alternately supplemented to form long nicked dsDNA. The HCR products can be recognized by TFO-FAM and form triplex DNA. So the TAMRA and FAM are brought closer together by the formation of triplex DNA, resulting in a FRET process and a dramatic decrease in the fluorescence intensity at 520 nm.

## Materials and methods

### Materials and chemicals

All oligonucleotides were synthesized and purchased from Sangon Biotech Co., Ltd. (Shanghai, China), the sequences were listed in Table 1. Oligonucleotides were dissolved in PBS buffer solution (pH 6.0, 10 mmol L^−1^, 0.1 mol L^−1^ NaCl) and stored at 4 °C. Spermine was purchased from Aladdin reagent Co., Ltd. (Shanghai, China). Human serum was purchased from guangzhou hongquan biotech. Co., Ltd. (Guangzhou, China).

**Table 1:** Sequences of oligonucleotides used in this work.

Name	Sequence (5′ → 3′)
Target VP DNA (VP DNA)	TTG GTC AAT ACT CAG CGA ATG TCT TTT GAC A
Single-base mismatch DNA (MT1)^a^	TTG GTC AAT ACT CAG TGA ATG TCT TTT GAC A
Two-base mismatch DNA (MT2)^a^	TTG GTC AAT CCT CAG CGA AGG TCT TTT GAC A
Three-base mismatch DNA (MT3)^a^	TTG GTC ACT ACT CAG AGA ATG TCG TTT GAC A
Non-complementary DNA (Non)	ATG CTG CTC AGC CAG CAC TCG CCC CTT CAT C
Molecular beacon (MB)	AAG AGA GAA CAA GCA GGA ATT TGT CAA AAG ACA TTC GCT GAG TAT TGA CCA AAA TTC CTG
Hairpin probe 1 (H1)	AAT TCC TGC TTG TTC TCT CTT AAG AGA AAG AGA GAA CAA GCA—TAMRA
Hairpin probe 2 (H2)	AAG AGA GAA CAA GCA GGA ATT TGC TTG TTC TCT CTT TCT CTT
Triplex-forming oligonucleotide (TFO)	TTC TCT TTC TCT CT—FAM

^a^Mismatch bases are underlined.

### Apparatus

Fluorescence intensity performed with an F-7000 fluorescence spectrophotometer (Hitachi, Japan). The excitation wavelength used was 480 nm, and emission spectra of 490–650 nm were recorded. Gel images were obtained using the ChampChemi Gel Image Analysis System (Beijing, China). Circular dichroism (CD) spectroscopy was analyzed by Chirascan™ V100 spectropolarimeter of Applied Photophysics Ltd. (Britain).

### Detection of target VP DNA

To prepare the hairpin probes (MB, H1-TAMRA and H2), the oligonucleotides were dissolved in PBS (10 mmol L^−1^ PBS, 0.1 mol L^−1^ NaCl, pH 6.0), then heated in a water bath at 95 °C for 5 min and cooled at room temperature for at least 4 h. VP DNA was mixed with MB (50 nmol L^−1^) hairpin probe and heated at 85 °C for 5 min and cooled at room temperature for 25 min. H1-TAMRA (100 nmol L^−1^) and H2 (100 nmol L^−1^) were added to the above mixture and incubated at 37 °C for 2 h. TFO-FAM (250 nmol L^−1^) was added to the above reaction mixture and incubated at room temperature for 1 h. Finally, the reaction mixture was detected with a fluorescence spectrophotometer at 480 nm.

### Gel electrophoresis analysis

Agarose gel electrophoresis was used to evaluate the HCR process. Agarose gel concentration was 3%, electrophoresis buffer was 1 × Tris–borate-EDTA buffer (TBE, pH 8.0), and constant pressure electrophoresis was performed at 100 V for about 60 min at room temperature. The nucleic acid was stained by 4S Red Plus Nucleic Acid Stain.

### CD spectroscopy measurement

CD spectroscopy was used to evaluate the structure of DNA and to demonstrate the formation of triplex DNA. The conditions of the spectropolarimeter used for detection were as follows: the scanning range was 200–350 nm, the scanning speed was 10 nm/min, the path was 0.1 cm, and the response time was 1.0 s, and the bandwidth was 1.71 nm.

### Preparation of serum samples

Human serum was stored at − 20 °C and thawed at 4 °C before use. 10% human serum was prepared by mixing human serum with PBS buffer (v/v = 1:9) and then filtered with a microporous membrane filter (aperture 0.22 μm). Afterwards, 21.80 nmol L^−1^, 27.52 nmol L^−1^ and 38.88 nmol L^−1^ VP DNA serum samples were obtained by adding a certain amount of VP DNA to 10% serums.

## Results and discussion

### Scheme of the strategy

**Figure 1:**
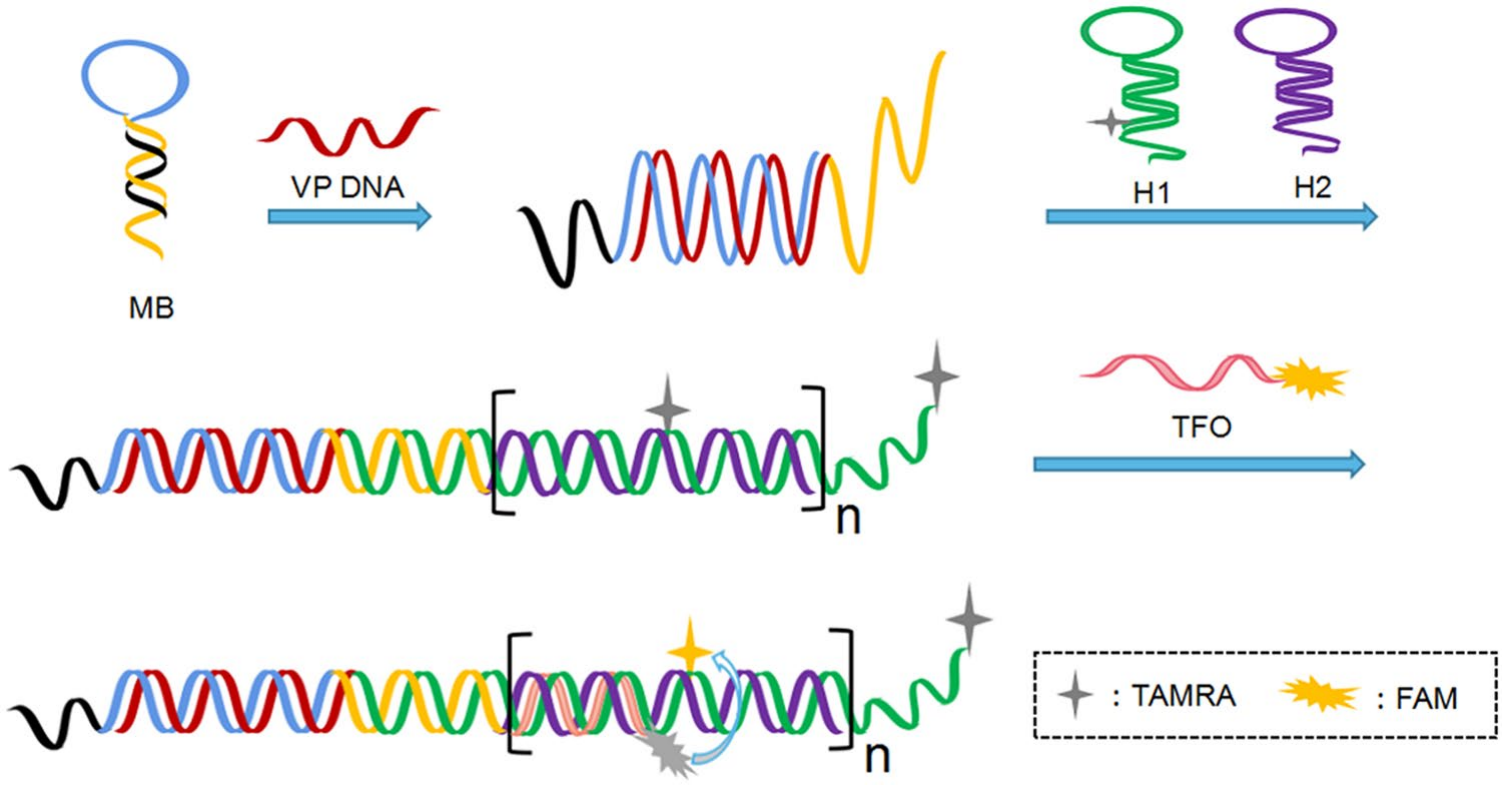
Schematic illustrating enzyme-free VP DNA detection based on HCR, FRET and triplex DNA.

As shown in Fig. 1, the single stranded DNA derived from the VP1331 gene was chosen as the target VP DNA. TFO and hairpin probes H1 are labeled with FAM and TAMRA, respectively. FAM and TAMRA are typical FRET pairs, FAM and TAMRA are fluorescent donors and fluorescent receptors, respectively. When target VP DNA is absent, HCR cannot be initiated. Resultantly, triplex DNA cannot form, and FAM is too far from TAMRA to FRET. In this case, the fluorescence intensity at 520 nm is strong under 480 nm excitation. With the addition of target VP DNA, the hairpin structure of MB is opened and the free end is released and hybridized with H1-TAMRA, the HCR reaction is triggered by the alternate supplementation of H1-TAMRA and H2 to produce dsDNA. After the addition of TFO-FAM, a triplex structure is formed between HCR products (H1-TAMRA/H2) and TFO-FAM. In this state, FRET pairs (FAM and TAMRA) approach each other, producing higher FRET efficiency. Therefore, quantitative detection of VP DNA can be achieved by detecting the decrease in fluorescence intensity of FAM at 520 nm.

### Feasibility of the strategy

**Figure 2:**
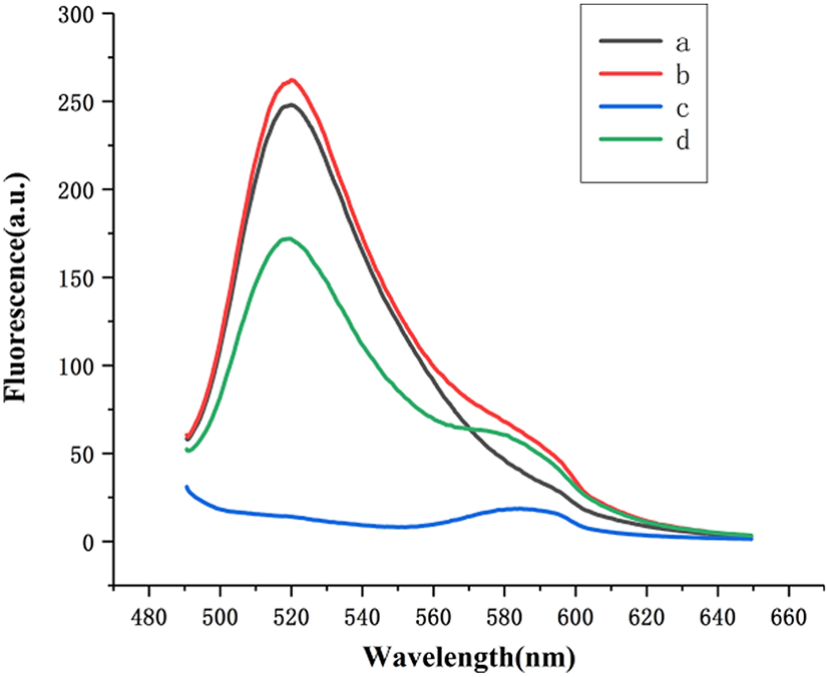
Fluorescence spectra of the sensing system (**a** MB + H1 + H2 + TFO; **b** VP DNA + MB + TFO; **c** VP DNA + MB + H1 + H2; **d** VP DNA + a). Experimental conditions: 50 nmol L^−1^ MB, 100 nmol L^−1^ H1 and H2, 250 nmol L^−1^ TFO.

To demonstrate the feasibility of the proposed strategy, the fluorescence signal of the system under different conditions were investigated. As shown in Fig. 2, there is no absorption peak at 520 nm due to the lack of TFO-FAM (curve c). When VP DNA was absent, HCR could not be triggered, resulting in the failure of the subsequent process, so the fluorescence intensity at 520 nm was very strong (curve a). In the absence of H1-TAMRA and H2, the double-strand and triplex structures could not form, resulting in FAM and TAMRA far away and FRET could not occur, so the fluorescence was also strong (curve b). However, target VP DNA addition to the mixture of MB, hairpin probe (H1-TAMRA + H2) and TFO-FAM reduced significantly the fluorescence intensity (curve d), indicating that the triplex DNA was formed and FRET occurred.

**Figure 3:**
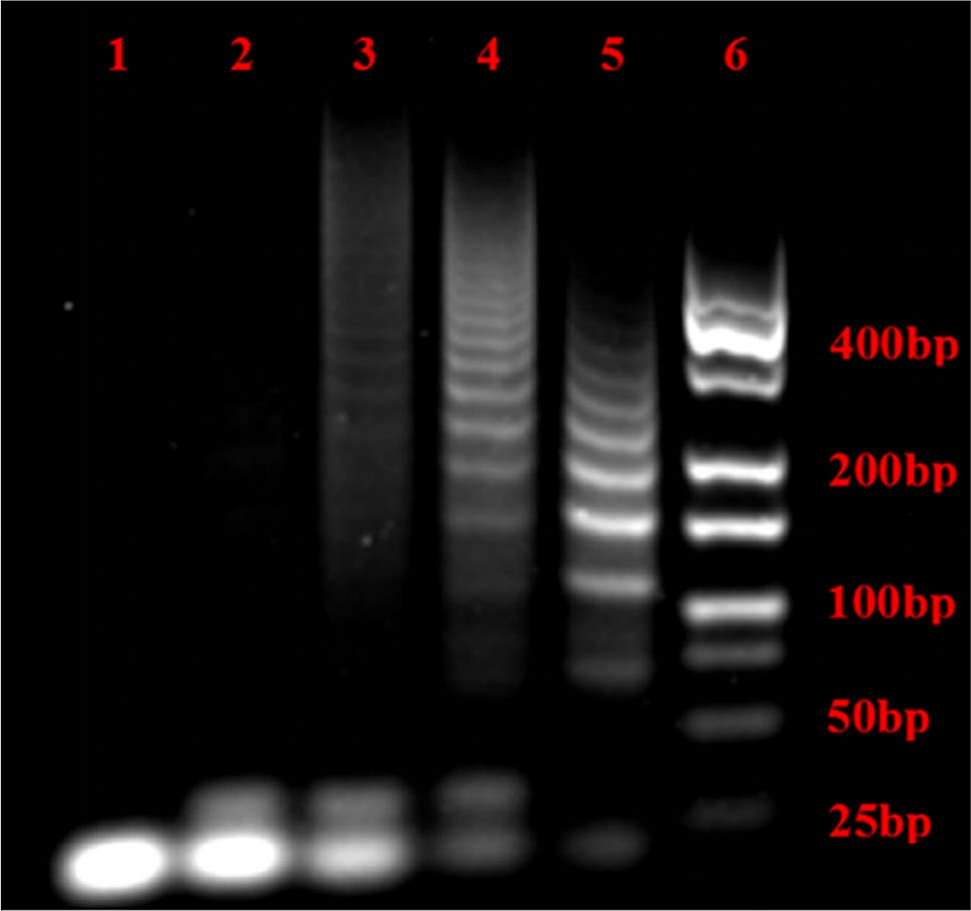
The gel electrophoresis images of different concentrations of VP DNA. Lanes 1: H1 and H2; lanes 2: MB, H1 and H2; lanes 3–5: different concentrations of target VP DNA (5, 50, 500 nmol L^−1^) with mixture of MB, H1 and H2; Lanes 6: the DNA Marker. Experimental conditions: 0.5 μmol·L^−1^ MB, 1 μmol·L^−1^ H1 and H2.

In addition, since target VP DNA activating HCR is prerequisite for building an amplification fluorescence sensing platform, the HCR process before and after the addition of the VP DNA was investigated with gel electrophoresis. As can be seen from Fig. 3, only one low-molecular-weight band in lane 1 (H1 + H2) and two low-molecular-weight bands in lane 2 (MB + H1 + H2) were observed, indicating that the hairpin probes in the mixed state were stable when VP DNA was missing, however, extended dsDNA appeared after addition of target VP DNA (lanes 3–5). The data showed that the HCR process occurred. Furthermore, it was found that the average molecular weight of HCR products was inversely proportional to the concentration of VP DNA, indicating that the hairpin probe consumption was positively related to VP DNA concentration.

**Figure 4:**
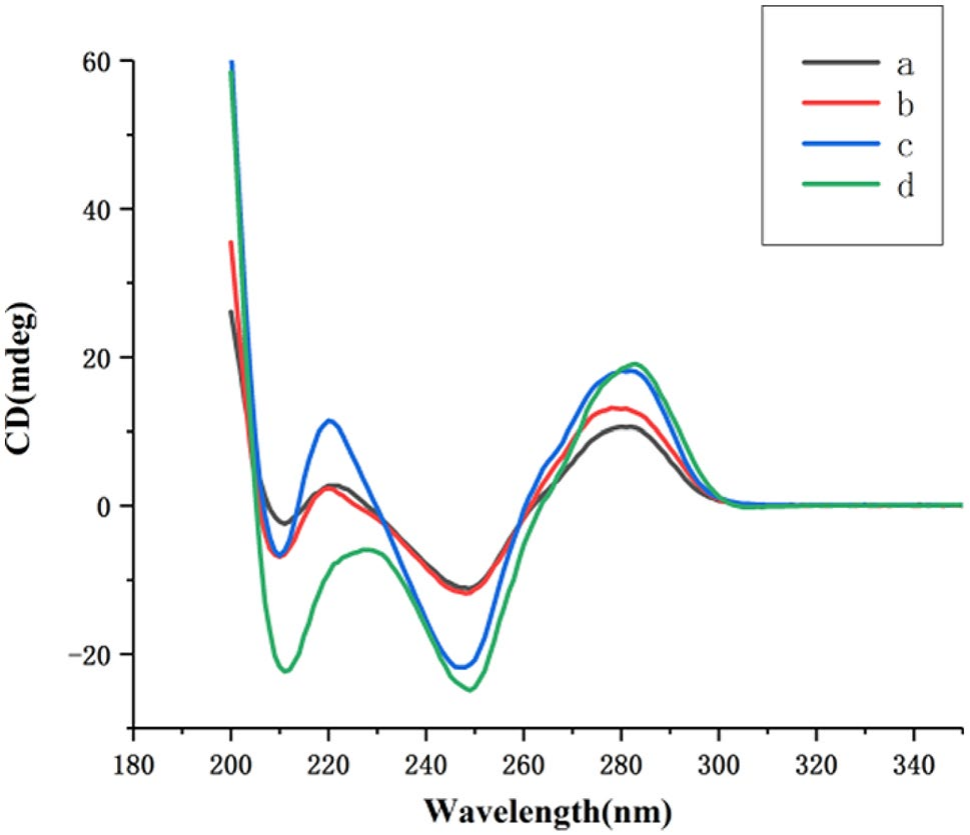
CD spectra of the sensing system (**a** VP DNA + MB + TFO; **b** MB + H1 + H2 + TFO; **c** VP DNA + MB + H1 + H2; **d** c + TFO). Experimental conditions: 5 μmol·L^−1^ MB, 10 μmol·L^−1^, H1 and H2, 25 μmol·L^−1^ TFO.

In order to demonstrate that HCR products form triplex DNA via binding with TFO, the DNA structures of different mixtures were determined by CD spectroscopy. As shown in Fig. 4, a positive peak at 278 nm and a negative peak at about 248 nm were related to dsDNA appearance in the CD spectrum. The values in curve c increased at 278 nm and about 248 nm as compared with the curve a and b, which was due to the long dsDNA of the HCR products. After the addition of TFO, CD spectroscopy (curve d) had a significant negative peak at 210 nm, which was an indicator of triplex DNA^29^. The data revealed that the strategy was feasible.

### Optimization of experimental conditions

**Figure 5:**
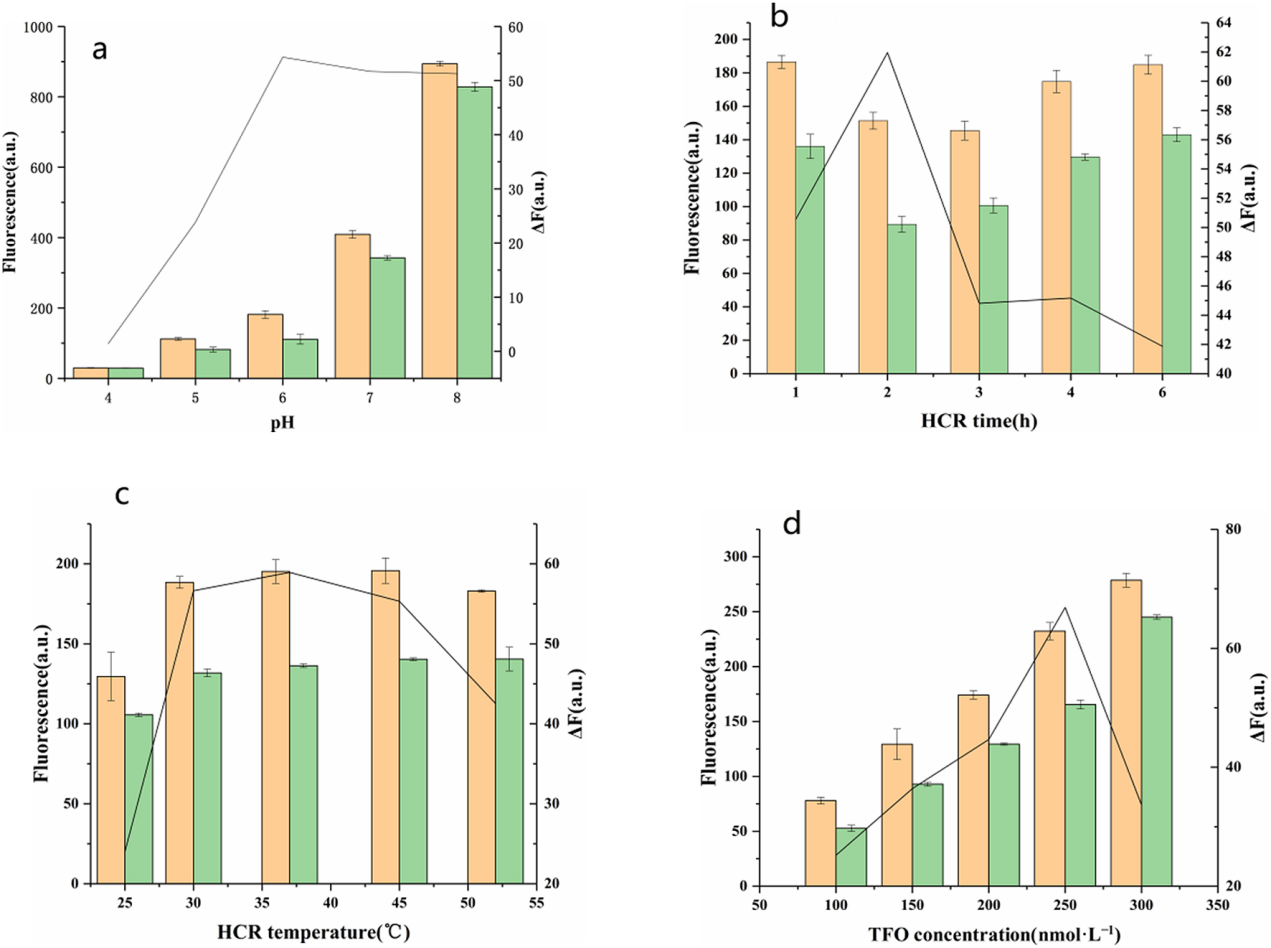
Effects of (**a**) the pH of PBS buffer, (**b**) reaction time of HCR process, (**c**) reaction temperature of HCR process and (**d**) the concentration of TFO on the performance of the sensing system. Red columns: control experiments; Blue columns: with 50 nmol L^−1^ of VP DNA. Black lines represent the ΔF at different conditions. The error bars represent the standard deviations of three replicates. Experimental conditions: 50 nmol L^−1^ MB, 100 nmol L^−1^ H1 and H2, 250 nmol L^−1^ TFO.

In order to maximize the response signal of the proposed strategy, various conditions were optimized: (a) the pH of PBS buffer, (b) the reaction time of HCR process, (c) the reaction temperature of HCR process and (d) the concentration of TFO. Their effects on the change of fluorescence intensity (∆F) were estimated with the equation ∆F = F_blank_ − F_target_, F_blank_ represented the fluorescence intensity of the mixture without VP DNA, F_target_ denoted the fluorescence intensity of the mixture in the presence of 50 nmol L^−1^ VP DNA. Data presented in Fig. 5 showed that the best results were observed under the following experimental conditions: (a) the pH of PBS buffer was 6.0, (b) the reaction time of HCR process was 2 h, (c) the reaction temperature of HCR process was 37 °C and (d) the concentration of TFO was 250 nmol L^−1^ .

### Sensitivity and selectivity of the proposed strategy

**Figure 6:**
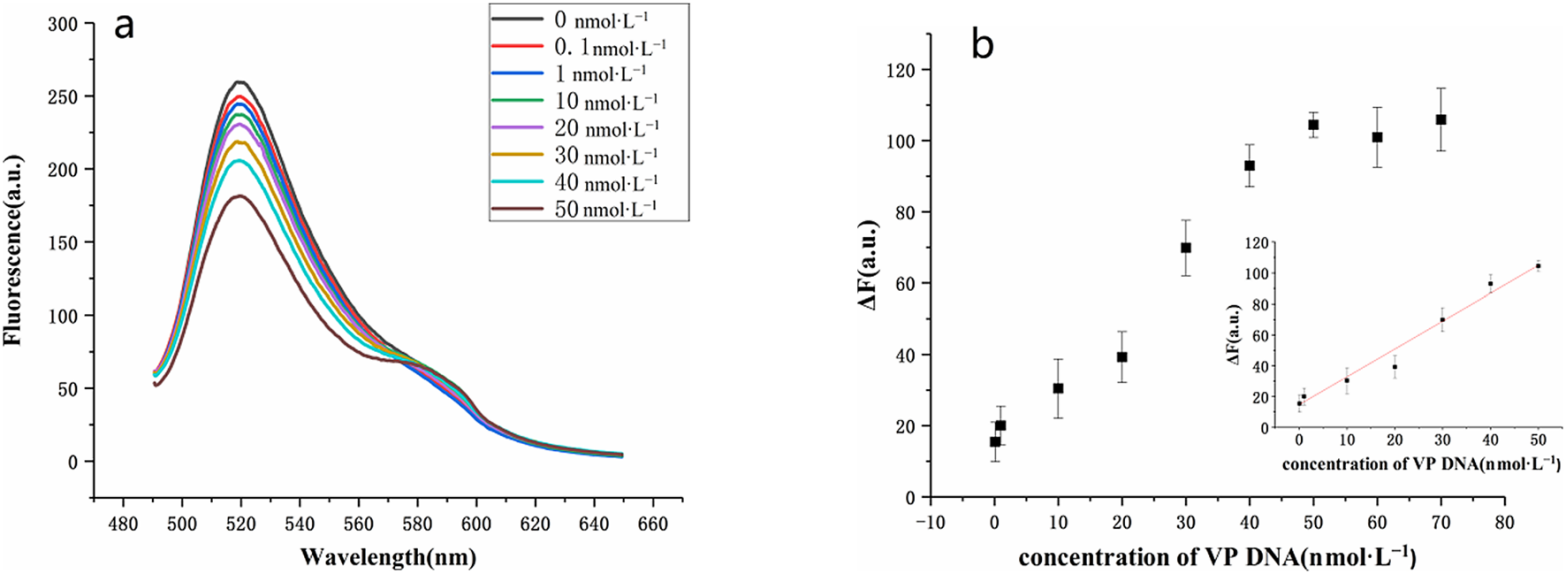
(**a**) Fluorescence spectra of the sensing system in the presence of various concentrations of VP DNA. (**b**) Plots of fluorescence quenching versus the concentration of VP DNA. Inset shows the calibration curve for VP DNA concentrations ranging from 0.1 to 50 nmol L^−1^. The error bars indicate the standard deviations of three measurements.

The sensitivity of the strategy was investigated with different concentrations of VP DNA (0.1–50 nmol L^−1^) under optimal conditions. Figure 6 showed that the fluorescence intensity at 520 nm gradually decreased with the increase of VP concentration, but gradually increased at 590 nm (a). As shown in, with the increase of target VP DNA concentration, fluorescence quenching value at 520 nm gradually increased (Fig. 6b). Meanwhile, the fluorescence quenching value was proportional to the concentration of target VP DNA during the range from 0.1 to 50 nmol L^−1^ with a linear regression equation of ∆F = 1.83C + 13.61 (R^2^ = 0.980, C represents the concentration of VP DNA (nmol L^−1^), ∆F denotes the change of fluorescence intensity) and a detection limit of 35 pmol L^−1^ (N = 3, RSD = 5.7%). HCR amplification and triplex DNA assembly were the reasons for the high sensitivity of this method.

**Figure 7:**
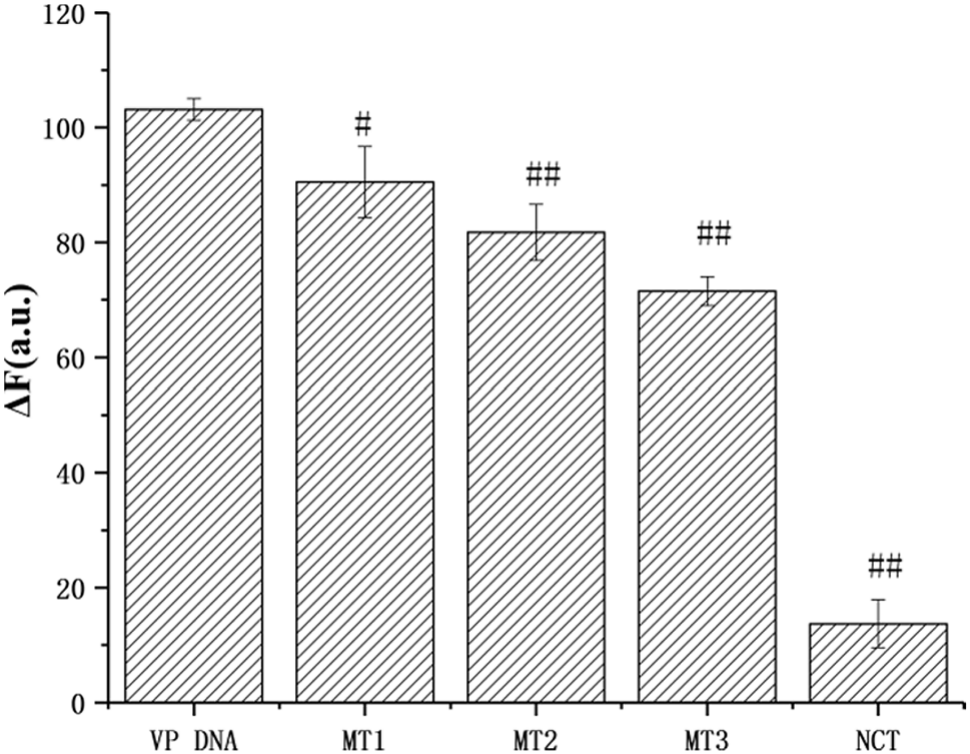
Fluorescent response at 520 nm for target VP DNA, MT1, MT2, MT3 and NCT. Experimental conditions: 50 nmol L^−1^ MB, 100 nmol L^−1^ H1 and H2, 250 nmol L^−1^ TFO, the concentrations of the above DNA are all 50 nmol L^−1^. Compared to VP DNA, ^#^p < 0.01, ^##^p < 0.001.

The selectivity of the strategy was studied with different initiators, including single base mismatched (MT1), two base mismatched (MT2) and tree base mismatched (MT3) VP DNA sequences and non-complementary DNA (NCT) (Fig. 7). The addition of NCT did not cause significant changes of fluorescence intensity, while the addition of VP DNA significantly increased the fluorescence quenching value. In addition, the fluorescence quenching value decreased with the increase of base mismatch. These results suggested that the addition of non-complementary DNA failed to open the hairpin structure, and that the ability to open the hairpin structure and triggering HCR decreased with the increase of mismatch bases. Compared with VP DNA, MT1, MT2, MT3 and NCT all showed significant differences (p < 0.05 or p < 0.01). So there seems to be that specific sequence of initiator is crucial for HCR initiation, and the HCR based approach has potential in single nucleotide polymorphism (SNP) analysis.

### Application

In order to verify the feasibility of this method for the direct quantitative determination of VP DNA in complex biological fluids, VP DNA in human serum was analyzed. Different concentrations of target VP DNA were added to the 10% serum (v/v) for detection according to this proposed method. The results are shown in Table 2. Data showed that this proposed method was feasible to detect target VP DNA in biological samples.

**Table 2:** Detection of VP DNA spiked in serum by the proposed method (n = 3).

Serum samples	Added (nmol L^−1^)	Found (nmol L^−1^)	Recovery (%)	RSD (%)
Sample 1	21.80	22.20	103.1	6.1
Sample 2	27.52	28.23	104.9	5.1
Sample 3	38.88	39.67	101.3	4.5

## Conclusion

In conclusion, an enzyme-free fluorescence sensitive detection platform based on HCR amplification, FRET and triplex DNA was constructed for sensitive and selective detection of VP DNA. In the presence of VP DNA, the MB hairpin structure is opened to trigger the HCR process to produce dsDNA. When TFO-FAM was added, TFO-FAM hybridized with the HCR products to form a triplex DNA, and the donor and receptor on TFO-FAM and H1-TAMRA are close to each other, resulting in a FRET process. The value of fluorescence quenching was used to quantitatively detect target VP DNA with a limit of detection of 35 pmol L^−1^. This method has a broad application prospect in the field of DNA diagnosis and clinical analysis.
